# InferAMP, a python web app for copy number inference from discrete gene-level amplification signals noted in clinical tumor profiling reports

**DOI:** 10.12688/f1000research.19541.3

**Published:** 2019-09-26

**Authors:** Paraic A. Kenny

**Affiliations:** 1 Kabara Cancer Research Institute, Gundersen Medical Foundation, La Crosse, WI, 54601, USA; 2 Department of Medicine, University of Wisconsin-Madison, Madison, WI, 53705, USA

**Keywords:** cancer, genetic testing, copy number variation, gene amplification, oncology, targeted therapy

## Abstract

As somatic next-generation sequencing gene panel analysis in advanced cancer patients is becoming more routine, oncologists are frequently presented with reports containing lists of genes with increased copy number. Distinguishing which of these amplified genes, if any, might be driving tumor growth and might thus be worth considering targeting can be challenging. One particular issue is the frequent absence of genomic contextual information in clinical reports, making it very challenging to determine which reported genes might be co-amplified and how large any such amplicons might be. We describe a straightforward Python web app, InferAMP, into which healthcare professionals may enter lists of amplified genes from clinical reports. The tool reports (1) the likely size of amplified genomic regions, (2) which reported genes are co-amplified and (3) which other cancer-relevant genes that were not evaluated in the assay may also be co-amplified in the specimen. The tool is accessible for web queries at http://inferamp.org.

## Introduction

Focal somatic gene copy number changes are a widespread event in tumor evolution ^1^. Although these regions of amplification may be large, encompassing many hundreds of genes, typically only one or a small number of genes within the amplified regions are involved in driving tumor growth. Identification of the key driver genes within recurrent amplicons has led to the approval of some therapies that have changed clinical practice (e.g. anti-ERBB2 agents ^2^); however, targeting other amplified genes such as FGFR family members ^3, 4^, *EGFR* ^5^ or *KIT* ^6^ has frequently proved disappointing. Nevertheless, even some of the more negative trials include occasional strong responses, indicating that sub-populations of patients with amplification of these oncogenes may experience clinical benefit if they can be identified. 

With the goal of individualizing treatment for cancer patients, next-generation sequencing from tumor specimens is becoming widely adopted ^7^. In addition to somatic point mutations, several of these assays report copy number changes in assayed genes. Reports for physicians typically present a list of amplified genes without providing a genomic context, leaving physicians and molecular tumor boards to hypothesize which of the listed genes might be driver genes suitable for therapeutic targeting. Given the poor response rates that have often been observed in clinical studies with amplified genes (compared to targeting genes activated by point mutation or fusion), physicians are often appropriately cautious about deciding whether a reported amplified gene may be actionable. Thus, many patients are spared receiving ineffective therapies, but a subgroup of patients who may experience clinical benefit do not get that opportunity.

Here we provide an easy-to-use web tool for analyzing clinical genomics reports of amplified genes. It determines (1) the likely size of amplified genomic regions, (2) which reported genes are co-amplified and (3) which other cancer-relevant genes that were not evaluated in the assay may also be co-amplified in the specimen.

The primary goals are to allow healthcare professionals to determine whether the amplification region surrounding a particular oncogene is relatively small and lacking in other likely candidate cancer drivers (which may indicate increased likelihood that the analyzed gene is a driver) from larger amplicons with additional candidate driver genes (which would suggest a reduced probability that the reported gene is a driver). The approach was developed and most extensively tested to analyze the widely used Foundation One test (329 genes) provided by Foundation Medicine. The web tool also allows selection of assays from several other test vendors, as well as pasting of a custom gene list from any currently unsupported assays.

## Methods

### Implementation

InferAMP is written in Python 2.7 with Flask and implemented as a web service running on the Google App Engine ( http://inferamp.org). Additional supplied requirements are (1) the coordinates of genes in the human genome, ‘coordinates.txt’ (hg38, UCSC genome browser), (2) The gene list from the assay of interest, ‘foundationone.txt’ and (3) a file listing genes recurrently altered in cancer from COSMIC ^8^ (retrieved 9/18/2019), ‘cosmic.txt’. This list includes all 723 Tier 1 and 2 genes in the COSMIC gene census.

An html page with a single query window allows the user to enter a comma-delimited list of genes reported as being amplified. The entry is passed to the script and parsed into individual gene names. An error check is performed to confirm that all entered gene names correspond to gene names in the genome used. The entered genes are considered to be amplified, while the other genes in the assay are considered to be not amplified.

**Figure 1.**
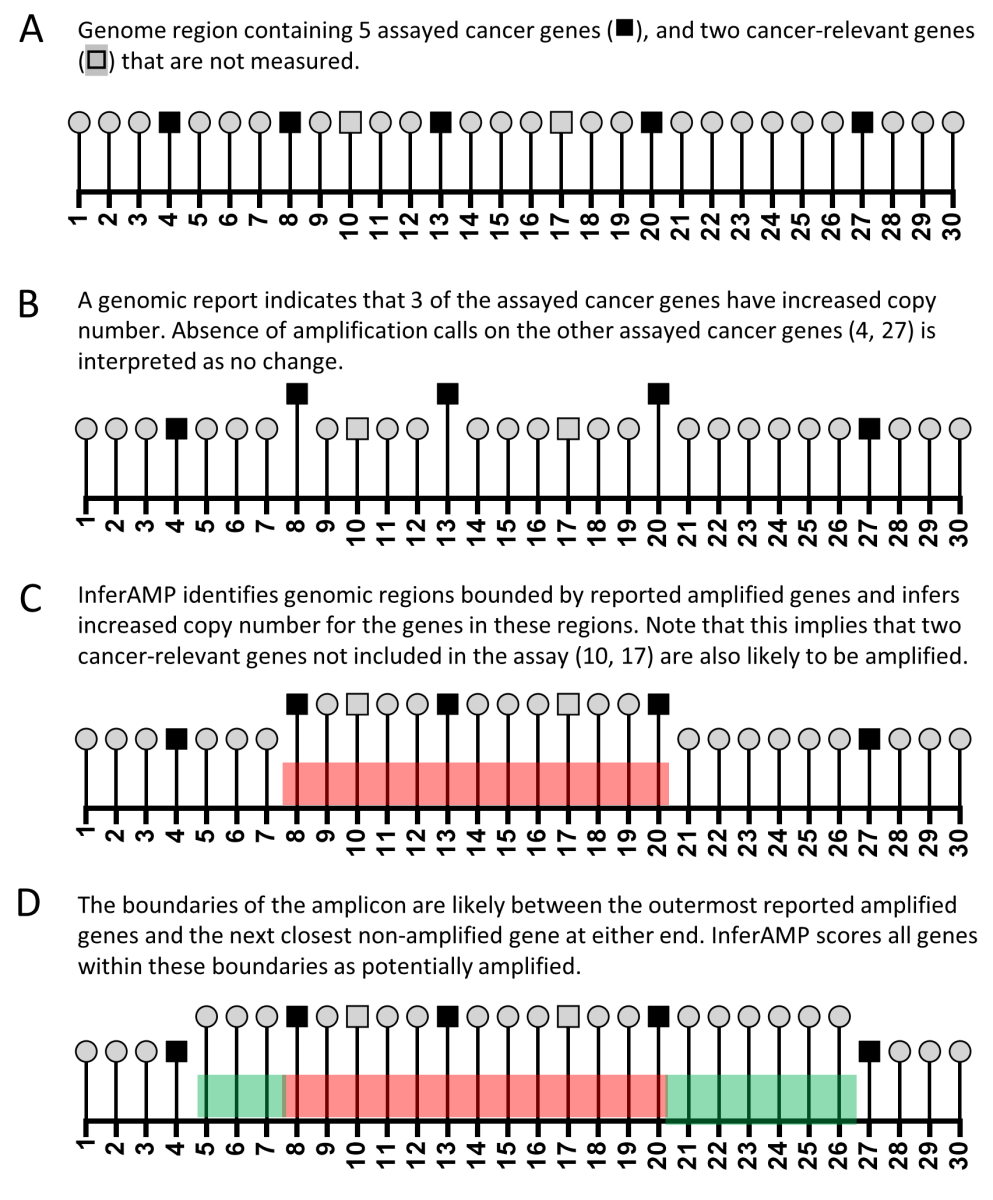
Schematic representation of amplicon boundary inference approach. ( **A**). Schematic diagram of a model genomic region with 30 numbered genes, which include a total of 7 cancer-relevant genes. ( **B**) Input scenario for algorithm: a clinical genomics report noting amplification of three genes in this region. ( **C**) Copy number inference for genes in regions bounded by reported amplified genes. ( **D**) Copy number inference for genes in the regions between the outermost reported amplified genes and the nearest reported non-amplified gene.

A simplified schema of how the algorithm works is presented in Figure 1, which depicts a chromosomal region containing 30 genes. Seven cancer-relevant genes are present, five of which are evaluated by the genomic assay ( Figure 1A). In this test example, three genes were reported to be amplified ( Figure 1B). Running the algorithm identifies these amplified genes (8, 13, 20) as well as the nearby assayed genes that are not reported amplified (4, 27). The algorithm considers all genes located between genes reported as amplified to also be amplified ( Figure 1C, red shaded region). Because not every gene is assayed, precisely delineating the boundaries of an amplicon is not possible. To address this, the algorithm determines the nearest non-amplified gene at each end of the amplicon and infers that the genes located up to, but not including that gene may be possibly amplified ( Figure 1D). The script then returns an html report page listing the entered genes, the amplicons into which they fall (in many cases, several discrete genes will be consolidated into a single amplicon), and also the other cancer-relevant genes within these regions that may be co-amplified with the reported genes. All genes reported include hyperlinks to that gene’s page on COSMIC. A checkbox on the form allows selection of “verbose” output which additionally includes (1) the complete list of assayed genes, (2) the identity of all genes in the inferred amplicons and (3) the identity of the assayed genes reported as non-amplified which are used to infer amplicon boundaries.

### Operation

InferAMP is accessed via a web browser and has been tested on commonly used browsers such as Chrome, Firefox, Internet Explorer and Microsoft Edge.

## Use cases

Three use cases taken from genomic reports of patients at our clinic are presented:

### Use case 1

A case of esophageal adenocarcinoma with eight reported amplified genes ( Table 1), which were resolved by InferAMP to four amplicons. The co-amplification of *FGF3*, *FGF4* and *FGF10* with *CCND1* (which is likely the driver gene in this amplicon ^9^) might indicate that consideration of FGFR inhibitors may not be helpful if these FGF genes are simply co-amplified passenger genes.

**Table 1.** Use case – Esophageal adenocarcinoma with reported amplification of *CCND1*, *MAP2K1*, *RICTOR*, *FGF10*, *FGF19*, *FGF3*, *FGF4* and *MCL1*.

Gene reported amplified (chromosomal location)	Number of potentially co-amplified genes (chromosomal region	Genes annotated as recurrently altered in cancer by COSMIC
*MCL1* (Chr1q21.2)	8 (Chr1p12–1q23.1)	*BCL9*, *PDE4DIP*, *ARNT*, *MLLT11*, *TPM3*, *MUC1*, *LMNA*, PRCC * *
*RICTOR* (Chr5p13.1), *FGF10* (Chr5p12)	75 (Chr5p13.2–5q11.2)	*LIFR*, *IL6ST*
*CCND1* (Chr11q13.3), *FGF19* (Chr11q13.3), *FGF4* (Chr11q13.3), *FGF3* (Chr11q13.3)	214 (Chr11q13.1–11q13.5)	*CCND1*, *NUMA1*
*MAP2K1* (Chr15q22.31)	224 (Chr15q15.1–15q22.31)	*B2M*, *USP8*, *MYO5A*, *C15ORF65*, *TCF12*, *MAP2K1*

### Use case 2

A case of soft tissue sarcoma with five reported amplified genes ( Table 2) which were resolved into three amplicons. In the absence of genomic context information, both *PDGFRA* and *KIT* might be considered as potentially druggable targets. The demonstration that these are likely co-amplified in a relatively small amplicon might provide further support to this hypothesis. Clinically, both targets are inhibited by imatinib, making joint targeting with a single agent feasible in this case.

**Table 2.** Use case – Soft tissue sarcoma with reported amplification of *KIT*, *PDGFRA*, *MDM2*, *RICTOR* and *FGF10*.

Gene reported amplified (chromosomal location)	Number of potentially co-amplified genes (chromosomal region)	Genes annotated as recurrently altered in cancer by COSMIC
*PDGFRA* (Chr4q12), *KIT* (Chr4q12)	97 (Chr4p15.31–4q12)	*SLC34A2*, *RHOH*, *PHOX2B*, *FIP1L1*, *CHIC2*, *PDGFRA*, *KIT*
*RICTOR* (Chr5p13.1), *FGF10* (Chr5p12)	75 (Chr5p13.2–5q11.2)	*LIFR*, *IL6ST*
*MDM2* (Chr12q15)	48 (Chr12q14.1–12q15)	*LRIG3*, *WIF1*, *HMGA2*, *MDM2*

### Use case 3

The third example is a breast cancer case from our clinic with three reported amplified genes ( Table 3). The report highlights one region on chromosome 5, and two regions on chromosome 7. The latter predicted amplicons share a nearby boundary at 7q22.3 suggesting the possibility that there is a regional amplification on 7q encompassing both sets of genes. In this case, *MET* was judged to be a possible driver amplicon, and the patient had a very strong response to a MET inhibitor ^10^.

**Table 3.** Use Case – Triple Negative breast cancer with reported amplification of *RICTOR*, *CDK6* and *MET*.

Gene reported amplified (chromosomal location)	Number of potentially co-amplified genes (chromosomal region)	Genes annotated as recurrently altered in cancer by COSMIC
*RICTOR* (Chr5p13.1)	44 (Chr 5p13.2–5p12)	*LIFR*
*CDK6* (Chr7q21.2)	190 (Chr 7q21.12–7q22.3)	*AKAP9*, *CDK6*, *TRRAP*, *CUX1*
*MET* (Chr7q31.2)	91 (Chr 7q22.3–7q32.1)	*MET*, *POT1*, *SND1*

## Discussion

We have described a straightforward tool to provide additional genomic context to aid interpretation of amplifications in somatic cancer sequencing reports. Use of this tool may aid decision-making by healthcare professionals about therapeutic options.

The method relies on the accuracy with which test vendors report gene amplification calls. Larger gene panels will also allow better resolution of amplicon boundaries. In testing, we identified a small number of cases in which two amplicons were inferred in very close proximity (e.g. *Use case 3*), which raises the possibility that the assayed gene between the two regions is erroneously not called as amplified. In cases with two or more closely co-located amplicons, users should consider that there is a strong possibility of a regional amplification encompassing both predicted amplicons. To assist with this determination, such cases are explicitly flagged in the reports. Future assays with larger number of genes or more sensitive amplification calling algorithms will likely permit more accurate refining of the boundaries of individual amplicons.

Because the coverage across the genome is somewhat sparse, refining the amplicon boundaries is more challenging than with a more high-density approach like SNP arrays. The primary purpose is to list genes that are potentially co-amplified with a gene identified by a test vendor as possibly actionable in order to allow healthcare professionals to gain further insight into the likelihood that the listed gene is truly the driver gene in that amplicon. Accordingly, we do not distinguish in the report between genes that are likely co-amplified (red genes, Figure 1) from the boundary region genes which are possibly co-amplified (green genes, Figure 1). In any case in which a healthcare professional might consider targeting a non-assayed gene predicted by this algorithm to be amplified (e.g. *LIFR* ^11^ in *Use case 2* and *Use case 3*), further clinical testing to directly confirm gene amplification would be warranted.

Although clinical NGS analysis of somatic changes in gene copy number is relatively new, comparison studies that have been performed against more established technologies such as gene-specific FISH probes and array comparative genomic hybridization have shown strong concordance ^12, 13^. Indeed, the ability to assess a large number of loci in parallel may be especially advantageous compared to current clinical FISH assays. Within the limits of our study (primarily focusing on the Foundation One assay and having no direct comparisons with alternative assay methodologies), we believe that our ability to easily infer contiguous amplicons involving multiple co-amplified assayed genes also supports the contention that these NGS assessments of copy number changes may be quite robust.

## Data availability

All data underlying the results are available as part of the article and no additional source data are required.

## Software availability

**Software available at:** http://inferamp.org.

**Source code available from:** https://github.com/paraickenny/inferAMP.

**Archived source code at time of publication:** http://doi.org/10.5281/zenodo.3457522 ^14^.

**License:** MIT License.

## References

[1] Zack TI Schumacher SE Carter SL: Pan-cancer patterns of somatic copy number alteration. *Nat Genet.* 2013;45(10):1134–40. 10.1038/ng.2760 24071852 PMC3966983

[2] Parakh S Gan HK Parslow AC: Evolution of anti-HER2 therapies for cancer treatment. *Cancer Treat Rev.* 2017;59:1–21. 10.1016/j.ctrv.2017.06.005 28715775

[3] Lim SH Sun JM Choi YL: Efficacy and safety of dovitinib in pretreated patients with advanced squamous non-small cell lung cancer with FGFR1 amplification: A single-arm, phase 2 study. *Cancer.* 2016;122(19):3024–31. 10.1002/cncr.30135 27315356

[4] Van Cutsem E Bang YJ Mansoor W: A randomized, open-label study of the efficacy and safety of AZD4547 monotherapy versus paclitaxel for the treatment of advanced gastric adenocarcinoma with FGFR2 polysomy or gene amplification. *Ann Oncol.* 2017;28(6):1316–24. 10.1093/annonc/mdx107 29177434

[5] Sepúlveda-Sánchez JM Vaz MA Balañá C: Phase II trial of dacomitinib, a pan-human *EGFR* tyrosine kinase inhibitor, in recurrent glioblastoma patients with EGFR amplification. *Neuro Oncol.* 2017;19(11):1522–31. 10.1093/neuonc/nox105 28575464 PMC5737732

[6] Hodi FS Corless CL Giobbie-Hurder A: Imatinib for melanomas harboring mutationally activated or amplified *KIT* arising on mucosal, acral, and chronically sun-damaged skin. *J Clin Oncol.* 2013;31(26):3182–90. 10.1200/JCO.2012.47.7836 23775962 PMC4878082

[7] Tan O Shrestha R Cunich M: Application of next-generation sequencing to improve cancer management: A review of the clinical effectiveness and cost-effectiveness. *Clin Genet.* 2018;93(3):533–44. 10.1111/cge.13199 29265354

[8] Tate JG Bamford S Jubb HC: COSMIC: the Catalogue Of Somatic Mutations In Cancer. *Nucleic Acids Res.* 2019;47(D1):D941–D7. 10.1093/nar/gky1015 30371878 PMC6323903

[9] Qie S Diehl JA: Cyclin D1, cancer progression, and opportunities in cancer treatment. *J Mol Med (Berl).* 2016;94(12):1313–26. 10.1007/s00109-016-1475-3 27695879 PMC5145738

[10] Parsons BM Meier DR Gurda GT: Exceptional Response to Crizotinib in an MET-Amplified Triple-Negative Breast Tumor. *JCO Precis Oncol.* 2017;1:1–6. 10.1200/PO.17.00070 35172503

[11] Hall BR Cannon A Thompson C: Utilizing cell line-derived organoids to evaluate the efficacy of a novel LIFR-inhibitor, EC359 in targeting pancreatic tumor stroma. *Genes Cancer.* 2019;10(1–2):1–10. 10.18632/genesandcancer.184 30899415 PMC6420790

[12] Seed G Yuan W Mateo J: Gene Copy Number Estimation from Targeted Next-Generation Sequencing of Prostate Cancer Biopsies: Analytic Validation and Clinical Qualification. *Clin Cancer Res.* 2017;23(20):6070–7. 10.1158/1078-0432.CCR-17-0972 28751446

[13] Su D Zhang D Chen K: High performance of targeted next generation sequencing on variance detection in clinical tumor specimens in comparison with current conventional methods. *J Exp Clin Cancer Res.* 2017;36(1):121. 10.1186/s13046-017-0591-4 28882180 PMC5590190

[14] Kenny P: paraickenny/inferAMP: inferAMP command line and web tool versions (Version v1.0.3). *Zenodo.* 2019 10.5281/zenodo.3457522

