# Supplementary material for: InferAMP, a python web app for copy number inference from discrete gene-level amplification signals noted in clinical tumor profiling reports
Source: F1000Res. 2019 Sep 26;8:807. Originally published 2019 Jun 6. [Version 3] doi: 10.12688/f1000research.19541.3 (PMC6777010; doi:10.12688/f1000research.19541.3)
Supplement: Peer review history

# Peer review: Reviewer response for version 3

**DOI:** 10.5256/f1000research.22814.r54388

**Published:** 2019-10-03

**Authors:** Andrew C. Nelson

**Affiliations:** 1 Department of Laboratory Medicine and Pathology, School of Medicine, University of Minnesota, Minneapolis, MN, USA

I appreciate the additional improvements that Dr. Kenny has made to the InferAMP tool and the edits made to clarify and strengthen the text. 

 This is a very useful tool for the molecular genetic pathology field and I fully approve of the manuscript.

I confirm that I have read this submission and believe that I have an appropriate level of expertise to confirm that it is of an acceptable scientific standard.

# Peer review: Reviewer response for version 3

**DOI:** 10.5256/f1000research.22814.r54389

**Published:** 2019-09-30

**Authors:** Oscar Krijgsman

**Affiliations:** 1 Division of Molecular Oncology and Immunology, Netherlands Cancer Institute, Amsterdam, The Netherlands

I have no further comments after reading the revised version from the author.

I confirm that I have read this submission and believe that I have an appropriate level of expertise to confirm that it is of an acceptable scientific standard.

# Peer review: Reviewer response for version 2

**DOI:** 10.5256/f1000research.21653.r51604

**Published:** 2019-08-15

**Authors:** Oscar Krijgsman

**Affiliations:** 1 Division of Molecular Oncology and Immunology, Netherlands Cancer Institute, Amsterdam, The Netherlands

The author describes a very straight-forward tool to infer possibly amplified genomic regions based on the amplification status of single genes from a targeted panel. InferAMP reports the genes that are likely co-amplified and in addition infers the size and the number of potentially co-amplified genes that were not assessed in the assay and outputs the COSMIC genes found in the genomic region.

 This tool is developed for oncologists to better understand the reported amplified genes in genomic context. The developed web-based tool is therefore important and will be the preferred way of using this tool for oncologists. It is also good to see the full code and data tables used are available on Github.

 InferAMP is a very easy tool to use and functions as advertised. The associated manuscript is well readable and explains the functionality satisfactory. However, after reading the manuscript and testing InferAMP I have a few questions that need to be answered.

 Points to be addressed:

1. The web-based tool is now only suitable for FoundationOne assays. Although this is very useful for many oncologists not all institutes use this assay. I am aware that the the command-line version of the tool has the possibility to provide different genes set and therefore works with additional assays. However, a command-line version will not be suitable for oncologists. Additional functionality of the web-based would greatly improve the usability of InferAMP.
2. The output of InferAMP includes the genes that are possibly co-amplified and the genes in the amplicon that are mentioned in the COSMIC database. In addition, the results mention the potentially co-amplified genes (for example 214 genes when running CCND1, FGF3, FGF4 and FGF19, Use case 1). A list of these genes is currently not available. It would be useful to output these lists of genes in addition to the COSMIC as not all targetable genes will necessarily be in the COSMIC list.
3. Currently the COSMIC list is used to provide an additional rationale to prioritize genes and identify genes suitable for targeting. The rationale for using this list is not provided in the text. A little more background and explanation for choosing this list would be helpful. In addition, which COSMIC list is used exactly? COSMIC cancer census list ^1^ or all genes mentioned in COSMIC?

 Points for consideration but not necessary for this manuscript:

1. Furthermore, I was wondering whether germline CNVs could affect the results of targeted panels, especially since patient-matched blood reference samples are not often used. If so, would it not be nice to add CNV data from for example the Database of Genomic Variants ( http://dgv.tcag.ca)? This could identify amplifications that are not somatic but merely ‘normal’ differences between individuals (CNVs).

I confirm that I have read this submission and believe that I have an appropriate level of expertise to confirm that it is of an acceptable scientific standard, however I have significant reservations, as outlined above.

## References

[1] : The COSMIC Cancer Gene Census: describing genetic dysfunction across all human cancers. *Nat Rev Cancer*.18(11) : 10.1038/s41568-018-0060-1 696-705 10.1038/s41568-018-0060-1 30293088PMC6450507

# Response

**Published:** 2019-09-24

**Authors:** Paraic Kenny

**Affiliations:** Gundersen Medical Foundation, USA

I greatly appreciate the thoughtful and supportive feedback and suggestions. These have been very helpful in revising the manuscript.

Major points:

1. We have added gene lists corresponding to seven additional cancer genomic reports (Foundation CDx, Trusight, Tempus, Caris etc.). We have also added a text box into which users can paste custom lists of genes for other unsupported assays.

2. We have added a new checkbox to the input page allowing users to request “verbose” output. If this is selected, all genes in the inferred amplicon are listed (i.e. not just the COSMIC genes).

3. We used the full COSMIC cancer gene census list (723 Tier 1 and 2 genes). The goal is to indicate to users where other potentially cancer-relevant genes are likely co-amplified with the reported genes so that users may consider whether the reported gene is truly the cancer driver in that region.

Minor Points:

1. Germline CNVs are an interesting question, but beyond the scope of the current project. This is a problem best left to the developers/interpreters/reporters of the various genetic assays. Our goal has been to provide an easy to use tool with which to assess genes reported as amplified after having passed some QC by commercial testers or clinical labs.

# Peer review: Reviewer response for version 2

**DOI:** 10.5256/f1000research.21653.r50604

**Published:** 2019-07-11

**Authors:** Andrew C. Nelson

**Affiliations:** 1 Department of Laboratory Medicine and Pathology, School of Medicine, University of Minnesota, Minneapolis, MN, USA

In this manuscript, Dr. Kenny describes a useful and straightforward web-based informatics tool that enables genetics professionals to rapidly and potentially more accurately infer genomic relationships of copy number alterations (CNA) disparately reported as individual genes in clinical genomics reports. This will aid both clinicians in working/teaching conferences (such as molecular tumor boards) and translational researchers reviewing archived clinical data.

 The rationale for the tool and the overview of the method is clear. The web interface is easy to use and provides a clear result report; code is available at Github.

 I have several points for improvement, clarification, and commentary on the manuscript and the software tool:

 Major Considerations 

1. I recommend that the assayed genes without amplification which are used by the algorithm as boundary genes be explicitly reported in the interface. In Fig 1D, these are represented as genes 4 and 27; as a minor point, I believe the legend for Fig 1D needs to be updated to be congruent with the figure. In the current interface report, the region of potential contiguous amplifications are only reported as cytobands. Specifically reporting the identity of these boundary genes would help genetics professionals to more precisely quality control the interpretation of both the original genomics report and the interface output.
2.  The tool is currently static configured for FoundationOne (F1); it would be beneficial to configure the web interface to allow (perhaps through a drop down menu) selection of other reasonably common cancer genomics reports which offer CNA data such as: Caris, Tempus, and the Illumina products TST170 and TSO500 (which are being deployed by some academic laboratories for clinical testing). Ultimately, an option to input a text file with HGVS gene names would be beneficial.
3.  I would suggest an expanded introduction or discussion about the status of clinical utility for gene amplification from comprehensive genomic profiling NGS assays. For example, only ERBB2 amplification is specifically included in the FDA premarket approval of the F1 assay ( https://www.accessdata.fda.gov/scripts/cdrh/cfdocs/cfpma/pma.cfm?id=p170019, accessed 07/10/2019). The most common diagnostic approach for gene amplification in clinical practice and clinical trials is FISH, frequently normalized to the centromeric copy number of the same chromosome on which the gene of interest resides (for example, see references 2-5 of the manuscript). There are, of course, significant pitfalls in the use of FISH as a longer established clinical standard, but I am not immediately aware of comprehensive accuracy assessments for somatic CNA analysis across broad numbers of genes by NGS, particularly in unpaired tests (i.e. no patient-specific normal sample for comparisons). A more in-depth review of these considerations and limitations in the introduction or discussion would equip readers to better interpret the expected output of datasets and any results generated using the tool.

Minor Comments and Commentary

1. Please clarify in the methods if the COSMIC Cancer Gene Census list in the tool has been filtered for type of alteration (i.e. SNV and small indel vs. gene rearrangement vs. CNA). Or is it the full 719 CGC list as referenced?
2. It may be valuable to consider cross-referencing the cytobands and/or cancer-associated genes within the predicted CNA region against regions/genes commonly copy number altered in pan-cancer analyses. For example: Beroukhim *et al*.[ Nature 2010 ^1^, Zack *et al*. Nature Genetics 2013 ^2^, Ohshima et al. Scientific Reports 2017 ^3^. Presenting this information in the report interface in future iterations would improve the quality and utility of the software tool.

 The ability to specifically annotate case results against disease-specific databases (i.e. TCGA projects) would also be valuable (see below as well).

 Commentary on use cases.

 The use cases provide a reasonable snapshot of how the tool functions and can be applied. Specifically, use case 1 highlights a common CNA in cancer (11q13.3) which has been described previously (Zack *et al*. Nature Genetics 2013 ^2^) and this specific amplicon is commonly seen in clinical genomics reports at our institution. The question of utility around FGFR inhibitors has arisen in our own molecular tumor boards based on the co-amplified FGF ligand genes, when in reality CCND1 is most likely the significant driver.

 Use case 2 highlights potentially actionable genes (KIT, PDGFRA) located in the same cytoband (4q12). However, the region of potential co-amplification crosses the Chr4 centromere (Chr4p15.31-4q12). It may be beneficial to further discuss how carefully to interpret amplicons which span both chromosomal arms. In this particular case, I infer that no reported amplified genes are present on 4p; but no boundary genes were analyzed centromeric to the PDGFRA locus. This seems worth a deeper discussion in the text.

 Use case 3 highlights an important point about potential drop out of amplification calls in NGS data. Dr. Kenny proposes that the entire amplicon on 7q is amplified, which is a reasonable hypothesis. It is important to note that specific bioinformatics pipelines and wet-bench library prep methods will not have equivalent analytic sensitivity/specificity for amplification calls for every captured gene. Case 3 is also interesting because the amplicon included CDK6, which is a target for FDA approved drugs in hormone receptor positive metastatic breast cancer. It might be interesting for Dr. Kenny to further comment on whether CDK inhibitors were considered less likely based on the triple negative hormone receptor status (PMID: 30038670 ^4^).

 I ran several of my own cases through InferAmp. Of interest, a case of high grade serous epithelial ovarian carcinoma is illustrative of the utility of the tool. This case had 8 separate gene amplifications reported by F1, including: KRAS, FGF23, FGF6, and CCND2 (all on Chr12p). The static clinical report indicated KRAS amplification was potentially relevant for MEK inhibitor therapy. Nevertheless, my molecular tumor board noted this amplification contig and noted that 12p is commonly amplified in high-grade serous carcinoma (TCGA); therefore it was unlikely to be a patient-specific driver alteration. The InferAmp quickly and accurately identified this amplicon, with the caveat that a potentially erroneous “break” was present at 12p13.1 (similar to Dr. Kenny’s use case 3).

 Finally, the final sentence of the manuscript cautioning that inferred co-amplified genes should be confirmed by CLIA-validated assays cannot be emphasized enough.

I confirm that I have read this submission and believe that I have an appropriate level of expertise to confirm that it is of an acceptable scientific standard, however I have significant reservations, as outlined above.

## References

[1] : The landscape of somatic copy-number alteration across human cancers. *Nature*.2010;463(7283) : 10.1038/nature08822 899-905 10.1038/nature08822 20164920PMC2826709

[2] : Pan-cancer patterns of somatic copy number alteration. *Nat Genet*.2013;45(10) : 10.1038/ng.2760 1134-40 10.1038/ng.2760 24071852PMC3966983

[3] : Integrated analysis of gene expression and copy number identified potential cancer driver genes with amplification-dependent overexpression in 1,454 solid tumors. *Scientific Reports*.2017;7(1) : 10.1038/s41598-017-00219-3 10.1038/s41598-017-00219-3 28377632PMC5428069

[4] : CDK4/6 inhibition in breast cancer: current practice and future directions. *Ther Adv Med Oncol*.2018;10: 10.1177/1758835918786451 1758835918786451 10.1177/1758835918786451 30038670PMC6050811

[5] : Identification of druggable cancer driver genes amplified across TCGA datasets. *PLoS One*.2014;9(5) : 10.1371/journal.pone.0098293 e98293 10.1371/journal.pone.0098293 24874471PMC4038530

# Response

**Published:** 2019-09-24

**Authors:** Paraic Kenny

**Affiliations:** Gundersen Medical Foundation, USA

I greatly appreciate the thoughtful and supportive feedback and suggestions. These have been very helpful in revising the manuscript.

Major:

1. We have added a new checkbox to the input page allowing users to request “verbose” output. In this case, the boundary genes are explicitly listed in the report.

2. We have added gene lists corresponding to seven additional cancer genomic reports (Foundation CDx, Trusight, Tempus, Caris etc.). We have also added a text box into which users can paste custom lists of genes for other unsupported assays. Because the accuracy of amplicon inference is related to the density/distribution of assayed genes across the genome, we added a comment on this to reports generated using panels with low gene numbers.

3. We have added a brief section to the discussion to discuss this, citing a couple of recent publications addressing the cross-comparison of NGS and FISH-based methodologies for CNA assessment.

Minor:

1. We used the 723 Tier 1 and 2 variants in the COSMIC gene census. This is now explicitly stated in the manuscript. While it may be reasonable to restrict the list to only those genes with known CNAs, we considered it best to use the broader list and trust the user to make the appropriate assessment. This avoids potentially missing situations where an oncogene that is typically activated by point mutation is activated by amplification under rare/unusual circumstances.

2. This is an excellent suggestion. We evaluated a number of CNA databases/datasets but implementing a straightforward and automated method for importing the data to annotate inferred amplicons in our reports was not feasible.

Use cases:

The point about drop-outs leading to inference of two adjacent amplicons where, in fact, there is just one true amplicon which contains an assayed gene not reported as amplified is an important one. As we now report the boundary genes (See point 1), we have implemented an additional check so that if a report contains two inferred amplicons which share a single boundary gene, this is explicitly flagged so that the user will be asked to consider the possibility that the “boundary gene” may be erroneously reported as non-amplified.
